# Cost‐Effectiveness of the Support and Services at Home (SASH) Program for Cardiovascular Risk Factors: A Community‐Based Approach to Healthy Aging in Place

**DOI:** 10.1111/1475-6773.70100

**Published:** 2026-03-10

**Authors:** Adam Atherly, Casey Doerner, Rong Yan, Jenn Schollmeyer, Liz Genge, Eline van den Broek‐Altenburg

**Affiliations:** ^1^ Department of Health Administration, College of Health Professions Virginia Commonwealth University Richmond Virginia USA; ^2^ Cathedral Square Corp. South Burlington Vermont USA; ^3^ Department of Radiology, Larner College of Medicine University of Vermont Burlington Vermont USA

**Keywords:** aging in place, cost effectiveness, wellness for older adults

## Abstract

**Objective:**

To estimate the cost effectiveness of the Support and Services at Home (SASH) program for health improvements associated with cardiovascular risk factors. Located in affordable housing units, SASH uses wellness approaches to prevent illness, manage chronic conditions and coordinate care delivery by connecting older adults and individuals with disabilities with community‐based services.

**Study Setting and Design:**

We calculated total quality‐adjusted life years (QALYs) gained from cardiovascular risk reduction and program costs using a Markov model.

**Data Sources and Analytic Sample:**

Data on changes in health status, health outcomes, and programmatic costs were drawn from SASH (primary) data sources from the statewide enrolled population in the original (Vermont) program. Data were collected from 2017 to 2023.

**Principal Findings:**

SASH reduced total cardiovascular risk factors including increases in appropriate medication use and reductions in systolic blood pressure. The cost per QALY gained ranged from $8344 to $4013 depending on gender and diabetes.

**Conclusions:**

SASH is a cost‐effective approach to improving the health of older adults and individuals with disabilities through a housing‐based community partnership. SASH is emblematic of the “wrong pocket” problem, so replication and funding of the model are challenging. For greater system efficiency and equity, finding ways to incorporate programs outside the healthcare system will be required.

## Highlights

- What is known on this topic?
  ○ Wellness interventions can aid in helping older adults successfully age in place.
  ○ The Support and Services at Home (SASH) is a community‐based program that has been shown to reduce the rate of growth of healthcare spending.
- What this study found?
  ○ The SASH program significantly improves cardiovascular risk factors in community‐based older adults, including significant changes in medication rates.
  ○ The SASH program extends life expectancy among the enrolled population by approximately 1 year with a very low cost per quality‐adjusted life year gained.

## 1 Introduction

Aging in place—defined as the ability of older adults to live safely and independently in their own homes and communities as they age—is increasingly recognized as a cornerstone of aging policy and practice. Surveys of older populations show that nearly 90% of older adults in the United States would prefer to remain in their current homes for as long as possible [1]. Aging in place has been linked to better mental health, enhanced quality of life, and the maintenance of social networks, while also reducing the demand for costly institutional care [2, 3]. It can also lead to substantial cost reductions for both individuals and health systems, particularly when combined with home‐ and community‐based services [4]. As demographic shifts increase the proportion of older adults, supporting aging in place is increasingly a practical necessity for sustainable long‐term care systems.

Successfully operationalizing programs to increase the ability of older persons to age in place is a challenge. Individuals often need support, which could include home modifications, accessible housing, or integrated care models. But one critical aspect of aging in place is the promotion of wellness in older populations. Promoting wellness in older adults is essential for maintaining independence, enhancing quality of life, and reducing the burden of chronic disease and disability [5]. As individuals age, they face increased risks of physical, cognitive, and emotional decline, yet wellness‐oriented interventions—such as physical activity, nutrition, social engagement, and preventive care—have been shown to mitigate these risks significantly [6]. Regular physical activity, for example, can improve mobility, reduce fall risk, and support mental health [7], while social connectedness is associated with lower rates of depression and cognitive decline [8].

One promising approach to promoting wellness in an older population is the Support and Services at Home (SASH) program. SASH is a community‐based initiative that integrates housing, healthcare, and social support to help older adults and individuals with disabilities live independently [9]. The SASH program uses a care team to conduct regular home visits, monitor the health of participants, develop individualized care plans, coordinate with local healthcare providers, and organize wellness activities [10]. Previous research has found that the SASH program has yielded positive health outcomes, particularly in preventive care. For instance, a 2017 survey reported that participants experienced improved management of chronic conditions such as diabetes and hypertension, fewer falls, and higher rates of immunizations and advance care planning among participants [11]. Similarly, a multi‐year evaluation found that the program significantly slowed the growth of Medicare and Medicaid expenditures, as well as specialist physician costs, particularly in urban areas [10, 12].

One of the keys to SASH is its ability to manage chronic diseases, the leading cause of both mortality and spending in older adults. Specifically, cardiovascular diseases (CVDs), particularly hypertension, pose a significant threat to older adults in the United States, leading to adverse health outcomes and substantial financial burdens. Nearly half of US adults have hypertension, a leading risk factor for CVD among older adults [13]. In 2021, adults receiving care for hypertension incurred an average of $2926 more in annual healthcare costs than those without hypertension, and projections suggest that by 2050, 61% of US adults will have some form of CVD, with associated costs expected to triple to $1.8 trillion [13].

Chronic disease management and prevention are widely recognized as cost‐effective strategies for reducing CVD risk. Community‐based interventions—such as blood pressure control [14], lifestyle modification [5], and medication adherence support [15]—have been shown to improve health outcomes and reduce costs, particularly among older adults and low‐income populations [16].

Low‐income populations face even greater challenges in managing CVD. Approximately 60% of excess heart disease in these groups is attributed to poverty‐related factors [17]. Among Medicare beneficiaries, 29% report cost‐related medication nonadherence, which is linked to increased morbidity, mortality, and healthcare expenditures [18, 19].

### 1.1 Intervention

SASH was started in Vermont in 2009 by a non‐profit housing provider, Cathedral Square Corporation (CSC) [20]. The purpose of SASH is to aid frail residents in affordable housing communities' access to and receive support to safely remain in their homes as they age. The program provides targeted support and in‐home services to participants, connects residents with community‐based support services, and improves health care coordination. Participants receive, at a minimum, access to a SASH coordinator and a wellness nurse that provide annual health and wellness assessments, individualized person‐centered care plans, individual nurse coaching, group programs focused on health and wellness, as well as care coordination with healthcare providers, including hospitals, primary care providers, and medical homes. All services offered have an evidence base for effectiveness, and the program has a relatively low rate of transition to long‐term care facilities (16%).

SASH is a unique combination of coordination and wellness teams embedded in affordable housing communities that provide support to manage chronic conditions and connect participants to health services and social supports [21]. SASH is situated in the social services sector in low‐income housing organizations but also has strong medical sector and public health sector connections. Public health sector connections include councils on aging and community mental health organizations. Medical sector connections include home health agencies, hospitals, medical homes, and primary care services. SASH staff work with health care providers to aid in successful hospital discharges, aid with medication management, and help coordinate services and create continuity of care. SASH also works extensively with the statewide Community Health Teams and with the State Department of Health to help the state meet population health goals around diabetes and hypertension.

In Vermont, there are 57 different SASH “panels” of participants. Each panel enrolls up to 100 participants. Although the program is based on affordable housing properties, Medicare beneficiaries living in the community are also eligible to enroll and have done so. Some panels are explicitly “mixed panels” of housing and community participants, while others are either entirely housing panels (“early panels”) or community ones (“community panels”). The program is delivered at the community level through the SASH panels, which are operated by housing host organizations. The host organizations vary widely and include properties funded by the Department of Housing and Urban Development, rural communities, and mobile home parks. Community‐based SASH sites are not in housing locations but instead utilize other local gathering places such as community centers.

SASH was designed as a system to integrate program silos: housing programs, health care programs, social service programs, and mental health programs. SASH aims to help older adults navigate disparate health care providers and services and fragmented social service programs. Each of these programs and services provides value individually, but without a centralized fiber to connect the ecosystem of care, not only is full value lost, but extra costs and pain are realized at an individual and systemic level.

The SASH program is an example of what is sometimes termed the “wrong pocket problem”. As a community‐based program, SASH aims to promote healthy behavior and improve access to care, offering the potential for significant health and economic benefits among vulnerable populations. But SASH does not realize the (potential) economic benefits—those benefits are accrued elsewhere in the system through reductions in utilization. Although the benefits of SASH are potentially hidden, the costs of the program are explicit. It is argued that the program benefits justify the costs, and that SASH is cost effective, or even cost‐saving. However, no prior research has evaluated its cost‐effectiveness. This study fills that research gap by studying the cost‐effectiveness of the SASH program in improving health as measured by QALYs and Life Years gained through reductions in cardiovascular disease risk factors.

## 2 Methods

The analytic approach for this study uses a Markov model to evaluate the cost effectiveness of the SASH program based on reductions in CVD risk factors. In this approach, actual outcomes are compared to a counterfactual based on simulated outcomes that would have been expected in the absence of the program. In this case, we observed changes in the health status of our population and compared that to a simulation where the intervention did not occur and thus the changes in health also did not occur. The population is older, but our simulation assumed no deterioration of health in the counterfactual to be conservative in our estimates.

### 2.1 Sample

The sample included a total of 6154 individuals, including 1395 males without diabetes, 581 males with diabetes, 3091 women without diabetes, and 1087 women with diabetes. Individuals were followed for an average of 5.4 years, which represents the average duration of individuals in the program before departing due to either transition to more intensive care settings or death.

Upon entry, participants are given health assessments. This health assessment is repeated annually as long as the individual remains in the program. The health assessment includes demographic data (age, gender) and as well as current medication usage, cholesterol rates, blood pressure, and reports of chronic illnesses, including diabetes. Participants were also asked about cigarette smoking and given a cessation program if appropriate. The health assessment is required for entry and thus everyone is included. The ending point of the data was the final health assessment.

### 2.2 Analytic Approach

A Markov model was constructed to calculate costs and outcomes following the model of Smith et al., 2019 [22]. A Markov model is a mathematical framework used to model systems that transition between a finite number of states over time with a simulation, where the probability of moving to the next state depends only on the current state. Using a cost‐utility framework, we compared gains in quality‐adjusted life years (QALYs) to changes in net costs. Our analysis only focuses on cardiovascular diseases (CVD).

**FIGURE 1:**
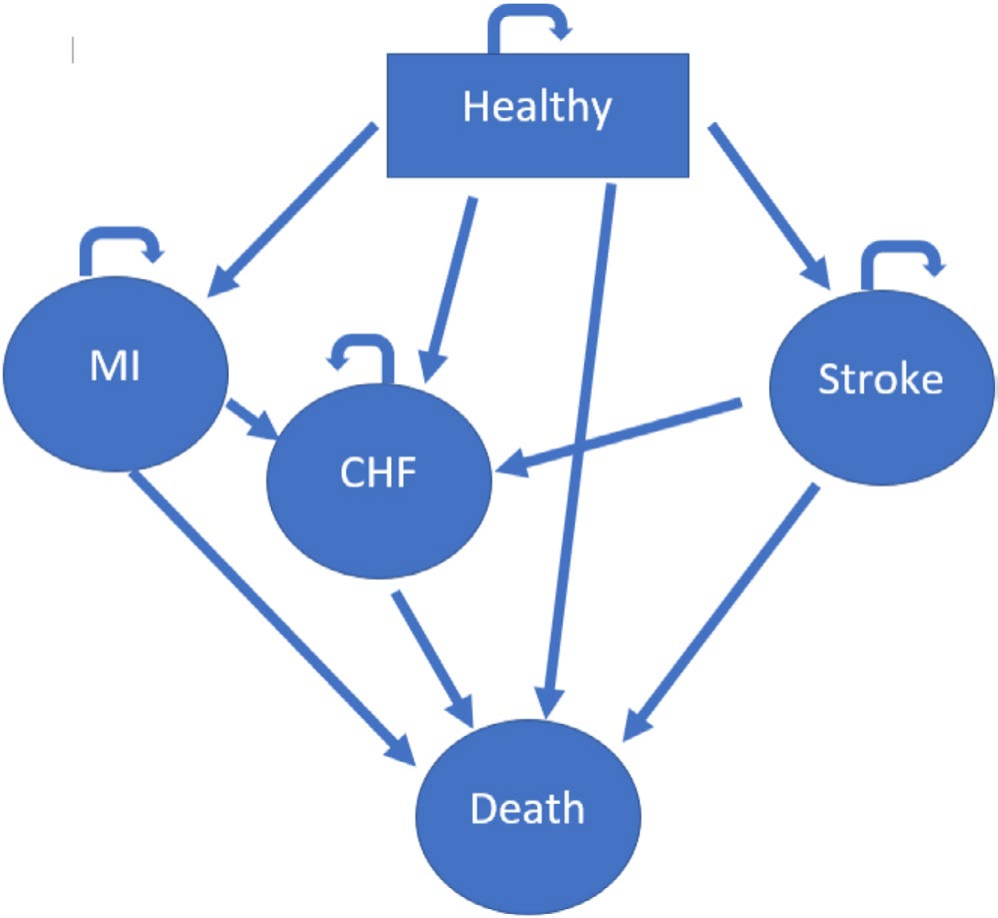
Markov model. Figure 1 illustrates a Markov state‐transition model in which individuals move among healthy, myocardial infarction (MI), congestive heart failure (CHF), stroke, and death states over discrete cycles, with death as an absorbing state. The arrows indicate allowed transitions, and the curved arrows denote state persistence across cycles.

Our Markov model includes five mutually exclusive states: normal health, acute myocardial infarction (MI), stroke (ischemic and hemorrhagic), post‐stroke, congestive heart failure (CHF), and death (Figure 1). All participants in the simulation begin in a state without CVD and then move to alternative states based on transitional probabilities, which are calculated using individual risk factors drawn from the health assessment as described above. In the model, individuals in the healthy state can remain in that state or progress directly to any of the other states, including death, based on the transition probabilities. Individuals cannot return to a healthy state once an adverse health event occurs. For both MI and stroke, individuals can either remain in that state or progress to either death or CHF. From CHF, individuals either remain in that state or progress to death. The time horizon is 20 years with a cycle length of 1 year. All costs and benefits were discounted at 3%. The transition probabilities were based on risk estimates drawn from the Framingham Heart Study [23, 24, 25, 26, 27]. Risk factors for the model included age, sex, total cholesterol, diabetes status, smoking status, systolic blood pressure (SBP), and use of hypertension medications (Table 1). All of these values were drawn from the health assessment.

**TABLE 1:** Base case and at‐risk input parameters.

Risk factors	Base male	Male diabetes	Base female	Female diabetes
Age when entering SASH (years)	66.2	66.6	68.9	67.9
Starting cholesterol (mg/DL)	162	180	195	173
Most recent cholesterol (mg/DL)	160	179	194	174
Change over time cholesterol (mg/DL)	−2.4	−0.86	−1.6	0.38
Starting SBP (mmHg)^a^	129	141	130	131
Estimated SBP (mmHg)	141	153	142	143
Using hypertension medication at baseline (%)^b^	6%	22%	14%	6%
Using hypertension medication at exit (%)	40%	60%	41%	68%
Smokers at baseline (%)	27%	17%	15%	14%
Smokers at exit (%)	24%	15%	13%	12%
Participants that quit smoking (%)	3%	2%	2%	2%
Sample size^c^	1395	581	3091	1087

^a^ SBP is systolic blood pressure.

^b^ Percentages represent the proportion of beneficiaries using hypertension medication at baseline.

^c^ Table provides baseline and follow‐up cardiovascular risk factor inputs used to parameterize the Markov model, stratified by sex and diabetes status, based on SASH participant health assessments. The total sample size is 6154.

The baseline values from the health assessment from SASH participants were converted to one‐year event probabilities based on the Framingham Health Study risk estimates. The intervention effect was based on observed changes in age, SBP, medication usage and smoking status, as shown in Table 2. The counterfactual was the assumption of no change in underlying risk factors. The model was stratified by sex and diabetes status, with different transition probabilities for each of the four groups. Diabetes was selected both because of the availability of inputs to calculate the transition probabilities and the relative frequency of diabetes. Framingham found that diabetes increased the CVD risk associated with other factors (e.g., smoking or high blood pressure), so the stratification should create more precise estimates.

**TABLE 2:** Transition probabilities for baseline non‐intervention males without diabetes.

From (to)	Healthy	Stroke	MI	CHF	Death
Healthy	0.9057	0.0070	0.0090	0.0100	0.0198
Stroke	0.0000	0.4053	0.0392	0.0488	0.1813
MI	0.0000	0.0000	0.5078	0.1230	0.3691
CHF	0.0000	0.0000	0.0000	0.4873	0.5127
Death	0.0000	0.0000	0.0000	0.0000	1.0000
Utility	0.85	0.65	0.70	0.60	0.00

*Note:* Table 2 exhibits annual transition probabilities between health states in the Markov model for baseline males without diabetes under the non‐intervention (counterfactual) scenario, along with the associated health state utility weights.

Costs were based on two different factors and represent health system costs. The first factor is SASH programmatic costs per panel, with an assumed participation of 100 members per panel (Table 3). Personnel costs included the full‐time site community health worker, the part‐time healthcare provider (a wellness nurse (RN)) plus part‐time supervisory and administrative support. Operating costs included travel time (for support staff), laptop computers, plus training and software licenses. These data were drawn from the SASH administrative records. Total direct costs were summed and then a 10% overhead cost was added to cover other indirect costs, including space.

**TABLE 3:** Program costs.

Item	Average panel costs
Personnel	
Community health worker^a^	$45,760.00
Wellness nurse^b^	$16,640.00
Supervisor/IM/TL^c^	$3900.00
Full administrative team per panel (59 panels)	$9149.00
Fringe (33%)^d^	$24,898.00
Total personnel cost	$100,347.00
Operating	
Travel 35 miles a week 52 weeks	$1183.00
Equipment 2 laptops	$3000.00
Supplies/materials	$3775.00
Bamboo health	$102.00
PHL	$1261.00
Relias training	$156.00
Phone plan	$600.00
Total operating cost	$10,077.00
Total direct charges	$110,424.00
Total indirect charges	$11,042.40
Total costs^e^	$121,467.00
Average cost per participant (100 participants per panel)	$1215.00

^a^ The average wage for community health workers is $22 per hour, with 40 h of work per week.

^b^ The average wellness nurse salary is $32 per hour, with 10 h of work.

^c^ Supervisor, Intermediate Manager, and Team Leader salaries are calculated as part‐time support at $25 per hour for 3 h.

^d^ Employee fringe benefits are calculated at a rate of 33% of the average wage.

^e^ Annual SASH program costs are estimated per panel and include personnel, operating expenses, and indirect costs, with the average per‐participant cost based on 100 participants per panel.

The second factor is expected healthcare costs. Healthcare costs were drawn from the literature [28, 29, 30, 31]. For each state, there is a cost associated with remaining in that state at the end of the year. When an individual transitions to a new state, there is a cost associated with that transition that represents the cost of the acute event. The individual then has a new steady state cost reflective of the average cost of the individual in that new state (e.g., a person will have a higher expected cost in the MI state than the healthy state). There is also a cost associated with the transition to death that represents the average age adjusted cost of mortality in the Medicare population [28]. Total costs were calculated by summing predicted healthcare costs over the 20‐year time horizon, discounted by 3%, plus the average cost per participant in the program.

QALYs were calculated based on utility data from previous research. QALYs are standard measures of health used in cost utility and cost effectiveness analysis and are a continuous measure from 1 (full health) to 0 (death). For our sample, we used a baseline utility value of 0.85 per healthy year (Table 2) [32], with QALYs declining to 0.70 for MI [33], 0.65 for stroke [34, 35] and 0.60 for CHF [36, 37]. We also calculated Life Years as an alternative measure [38]. For both QALYs and Life Years, we summed predicted values over the 20‐year time horizon, again discounted at 3%.

The perspective of the study is the health system. The challenge for SASH is that it is funded by direct allocation from the state with the expectation that the program offsets costs elsewhere in the health system. Thus, we use the health system perspective to see if the funding provided to SASH is justified by the QALY impact of the program.

## 3 Results

There were a number of notable changes in health during SASH program participation (Table 1). On average, males were approximately 66 years old at entry, while females were 68 years old. Overall cholesterol rates didn't change, but the percentage using hypertension medications increased by between 27% and 62%, depending on gender and diabetes status. The rate of smoking declined slightly.

The key outcomes in the model are the discounted net present value of Life Years (gained) and QALYs (gained) due to the reduction in risk from CVD. Looking at the model inputs (Table 2), there were two key health changes that notably changed the transition probabilities. First is the increase in the percentage of people using appropriate hypertension medication. For males, the percentage increased from 6% to 40% for participants without diabetes, while it increased from 22% to 60% for males with diabetes. Similarly, for females without diabetes, the percentage increased from 6% to 68% and from 14% to 41% for females with diabetes. This is also reflected in the superior SBP in participants as compared to standard projections of increases. There was also a slight decrease in smoking rates of approximately 2% for all groups. Because smoking is such a significant risk factor for MI, stroke and CHF, this relatively small effect has a significant impact on the outcomes.

Using the inputs from Table 1, we find that in the base case of a 66‐year‐old male without diabetes, program participants would be expected to have a discounted net present value of 8.8 Life Years and 7.1 QALYs remaining (Table 4). In contrast, a non‐participant would be expected to have a discounted net present value of 7.5 Life Years and 6.1 QALYs remaining. The program participants have higher total costs (Table 3), partly due to program costs ($1215 per participant per year), but largely due to higher total healthcare costs associated with the additional life years. The cost per QALY gained for males without diabetes in the SASH program as compared to non‐participation is $8344 and the cost per Life Year gained is $6418.

**TABLE 4:** Gains in QALYs, Life Years, costs and incremental cost effectiveness ratio.

	Base male	Male diabetes	Base female	Female diabetes
Expected QALYs and Life Years^a^				
Discounted QALY with intervention	7.1	4.9	7.9	6
Discounted QALY without intervention	6.1	4	7.3	5.1
Discounted Life Years with intervention	8.8	6	9.9	7.3
Discounted Life Years without intervention	7.5	4.9	9.1	6.2
Expected costs				
Cost with intervention	$55,179	$33,834	$35,324	$23,660
Total cost without intervention	$45,146	$26,562	$31,334	$19.23
Discounted cost with intervention	$43,580	$28,224	$27,497	$19.23
Discounted cost without intervention	$36,451	$22,790	$24,699	$16,000
Changes in QALYs, costs and ICER				
Gain in discounted QALYs^b^	1	0.9	0.6	0.9
Gain in discounted Life Years	1.3	1.1	0.8	1.1
Incremental cost	$8344	$6649	$4013	$4445
Cost per QALY gained	$8344	$7388	$6688	$4938
Cost per Life Year gained	$6418	$6045	$5016	$4041

^a^ QALYs denote quality‐adjusted life years.

^b^ Gains in discounted QALYs are calculated as the difference in QALYs between the intervention and non‐intervention scenarios.

The results for the base case for females are similar (Table 4). For a 69‐year‐old female without diabetes, SASH program participants would be expected to have a discounted net present value of 9.9 Life Years and 7.9 QALYs remaining. In contrast, a non‐participating female without diabetes would be expected to have a discounted net present value of 9.1 Life Years and 7.3 QALYs remaining. The SASH program participants again have higher total costs (Table 3), partly due to the same program costs ($1215 per participant per year), but largely due to higher total healthcare costs associated with the additional life years. The cost per QALY gained for female participants without diabetes in SASH as compared to non‐participation in SASH is $6688, and the cost per Life Year gained is $5016.

The results for participants with diabetes are again largely similar (Table 4). For male participants with diabetes, SASH participants gain a discounted net present value of 0.9 QALYs and 1.1 Life Years for an incremental cost per QALY gained of $6649. The gain in Life Years and QALYs is identical for female SASH participants, although the costs are lower at $4445. The cost per QALY gained is $6045 for male participants with diabetes and $4041 for female participants as compared to non‐participants (Table 3).

## 4 Discussion

This study provides evidence supporting the cost‐effectiveness of the Support and Services at Home (SASH) program. The models showed that the cost per QALY gained ranged between $8344 and $4041, depending on the sex and diabetes status of the participant. The gain in additional QALYs for males was approximately 1, while the gain in QALYs for females was lower, ranging between 0.6 and 0.9 QALYs. Although the precise threshold for an intervention to be considered cost effective varies, in the United States a threshold of $100,000 per QALY gained is often used [39, 40], indicating that SASH services are a cost‐effective approach to help older persons age in place. Indeed, at a cost per QALY gained of (at most) $8000, SASH is considered cost effective under any widely used benchmark.

Located in affordable housing units and the surrounding neighborhoods, SASH uses wellness approaches to prevent illness, manage chronic conditions, and coordinate care delivery by connecting older adults and individuals with disabilities with community‐based services.

It is likely that the analysis understates the true cost‐effectiveness of the SASH program. Overall, the average age of entry into SASH was 69 years old; age of exit was 77. Only 16% of SASH participants left the program for a higher level of care (i.e., Long Term Care (LTC)). For persons in this age range, on average 22% would be expected to enter LTC [41, 42]. This suggests that SASH may have reduced the rate of transition to LTC significantly. Given that the costs of a 2–4 months of LTC ($2540 per month) exceed the total increase in incremental costs associated with SASH [43], and that the average length of stay in LTC is 2.3 years [44], it is likely that the program significantly reduces total health system expenditures. For example, for the base case males, a reduction of 8.2 percentage points in the rate of LTC utilization would meet the breakeven threshold. Further research should study the question of changes in the rate of transition to LTC.

Our study has several important limitations. The model only allows a single cardiovascular disease event (i.e., a single stroke or MI). This will tend to produce a conservative result and underestimate the true cost effectiveness ratio. Our model also did not include additional prescription drug costs. However, the costs of standard hypertension medications are low and will not meaningfully impact the cost‐effectiveness ratio. We also did not include costs associated with survival, including living expenses. We drew data on healthcare expenditures from the literature rather than from actual costs. We also focus exclusively on CVD in this analysis because of the availability of well validated models to calculate transition probabilities. If the program benefits other health conditions, our estimates will underestimate the cost effectiveness of the program.

The goal of SASH is to improve health through care coordination and proactive care delivery. But SASH has the classic wrong pocket problem—a model with an evidence base suggesting a positive return on investment—yet the organizations that benefit from the spending reductions associated with SASH do not directly finance it, and SASH does not recoup the savings they create. SASH faces challenges in expanding, both within and beyond affordable housing into the community. Limited funding, particularly for wellness nurses, inhibits SASH from making greater inroads in existing service areas and prevents the model from being introduced in other regions. This highlights the challenge of providing appropriate funding for cost‐effective, community‐based wellness programs such as SASH.

## Funding

This work was supported by the Robert Wood Johnson Foundation, 80232.

## Conflicts of Interest

The authors declare no conflicts of interest.

## Data Availability

The data that support the findings of this study are available from the corresponding author upon reasonable request.
